# Investigación latinoamericana en falla cardiaca: análisis visual y bibliométrico de los últimos 20 años

**DOI:** 10.47487/apcyccv.v4i4.328

**Published:** 2023-12-27

**Authors:** Gisselle Batista Mendoza, Gustavo Alberto Giraldo Puentes, Enmanuel Rosero Palacios, Patrick Junior Brett Cano, Katerin Tatiana Ramírez Reyes, Cristhian Mauricio Zapata Valencia, Yohan Lilibeth Suarez Uribe, Alex Farith Reyes, Yelson Alejandro Acuña Picón-Jaimes

**Affiliations:** 1 Departamento de Medicina, Universidad del Magdalena, Santa Marta, Colombia. Universidad del Magdalena Departamento de Medicina Universidad del Magdalena Santa Marta Colombia; 2 Departamento de Medicina, Universidad de Caldas, Manizales, Colombia. Universidad de Caldas Departamento de Medicina Universidad de Caldas Manizales Colombia; 3 Departamento de Medicina, Fundación Universitaria Navarra, Neiva, Colombia. Departamento de Medicina Fundación Universitaria Navarra Neiva Colombia; 4 Departamento de Medicina, Universidad del Sinú, Cartagena, Colombia. Universidad del Sinú Departamento de Medicina Universidad del Sinú Cartagena Colombia; 5 Facultad de Medicina, Corporación Universitaria Rafael Núñez, Cartagena, Colombia. Corporación Universitaria Rafael Núñez Facultad de Medicina Corporación Universitaria Rafael Núñez Cartagena Colombia; 6 Grupo Investigación y Desarrollo en Cultura de la Salud, Universidad Tecnológica de Pereira, Pereira, Colombia. Universidad Tecnológica de Pereira Grupo Investigación y Desarrollo en Cultura de la Salud, Universidad Tecnológica de Pereira Pereira Colombia; 7 Departamento de Medicina, Fundación Universitaria Juan N Corpas, Bogotá, Colombia. Fundación Universitaria Juan N. Corpas Departamento de Medicina Fundación Universitaria Juan N Corpas Bogotá Colombia; 8 Departamento de Medicina, Universidad Simón Bolívar, Barranquilla, Colombia. Universidad Simón Bolívar Departamento de Medicina Universidad Simón Bolívar Barranquilla Colombia; 9 Programa de Doctorado en Salud, Bienestar y Bioética, Universidad Ramón Llul, Barcelona, España. Universitat Ramon Llull Programa de Doctorado en Salud Bienestar y Bioética Universidad Ramón Llul Barcelona Spain

**Keywords:** Insuficiencia Cardíaca, Enfermedades Cardiovasculares, Bibliometría, América Latina, Heart Failure, Cardiovascular Diseases, Bibliometrics, Latin America

## Abstract

**Objetivo.:**

Analizar de manera visual y bibliométrica la investigación latinoamericana en falla cardiaca en los últimos 20 años.

**Materiales y métodos.:**

Estudio bibliométrico que utilizó la base de datos Scopus. Se desarrolló una búsqueda no sistemática para la recopilación de datos, los cuales se analizaron a través de Bibliometrix, una herramienta del lenguaje de programación R.

**Resultados.:**

Se incluyeron 10 204 documentos en un período comprendido entre 2003 a 2023. De estos, el 66,9% (n=6824) correspondieron a artículos originales, seguidos por artículos de revisión (15,5%; n=1583). La colaboración internacional estuvo presente en el 38,41% (n=3919) de los artículos. Brasil se destacó con el mayor número de autores e instituciones prolíficas (70 y 60%, respectivamente), consolidándose como líder en la región, seguido por Argentina y México. Estos países también presentaron los documentos con mayor impacto y métricas más destacadas.

**Conclusiones.:**

Este estudio identificó un aumento significativo en la investigación sobre falla cardíaca en Latinoamérica durante las últimas dos décadas, siendo Brasil, Argentina y México los principales impulsores de esta tendencia. La colaboración extensa y sólida, principalmente con países de altos ingresos, parece ser fundamental para el impulso y avance de la investigación en esta área. La sistematización de datos y la terapia de resincronización son algunos de los temas de mayor interés en la actualidad.

## Abstract (en)

**Objective.:**

To visually and bibliometrically analyze Latin American research on heart failure in the last 20 years.

**Materials and methods.:**

A bibliometric study using the Scopus database. A non-systematic search was carried out to collect data, which were analyzed using Bibliometrix, a tool of the R programming language.

**Results.:**

A total of 10204 documents were included in a period between 2003 to 2023. Of these, 66.9% (n=6824) corresponded to original articles, followed by review articles (15.5%; n=1583). International collaboration was present in 38.41% (n=3919) of the articles. Brazil stood out with the highest number of prolific authors and institutions (70% and 60%, respectively), consolidating its position as leader in the region, followed by Argentina and Mexico. These countries also presented the papers with the highest impact and most outstanding metrics.

**Conclusions.:**

This study identified a significant increase in heart failure research in Latin America over the last two decades, with Brazil, Argentina, and Mexico being the main drivers of this trend. Extensive and strong collaboration, mainly with high-income countries, appears to be critical to the momentum and the advancement of research in this area. Data systematization and resynchronization therapy are some of the topics of greatest interest at present.

## Introducción

La falla cardiaca es uno de los fenotipos de enfermedad cardiovascular más prevalentes en Latinoamérica y en el mundo ^1^^,^^2^. Según datos de estudios clínicos, se estima que en el continente latinoamericano la incidencia de insuficiencia cardíaca puede ser de hasta, aproximadamente, 200 casos por cada 100 000 habitantes por año, y la prevalencia es cercana al 1% del total de la población ^3^. Esta condición afecta predominantemente a personas en edad funcional y en riesgo de muerte prematura (con una media de fracción de eyección del 36%) alrededor de los 60 años ^3^, lo que podría representar una prevalencia elevada de insuficiencia cardíaca sintomática entre los casos, afectando significativamente los resultados en salud y la calidad de vida ^4^.

Las brechas en investigación, específicamente en insuficiencia cardíaca en Latinoamérica, han sido un problema reportado por distintos autores ^5^^,^^6^. Esto implica el desarrollo y abordaje de un fenotipo de cardiopatía de acuerdo con características sociales, culturales, genéticas, demográficas y de salud propias de una región, que difieren de otras a nivel global ^7^. Esto, acompañado de barreras en el acceso oportuno a servicios de salud especializados y de alta calidad, además de la disponibilidad de medicamentos y herramientas tecnológicas que faciliten la implementación de medicina personalizada ^8^, influye de forma negativa en el pronóstico de esta enfermedad. La investigación traslacional y experimental podrían ser abordajes de investigación poblacional que den respuesta a interrogantes de interés en insuficiencia cardíaca, así como permitir el desarrollo de técnicas, el uso de biomarcadores y de intervenciones con resultados significativamente favorables ^9^. No obstante, para dar respuesta a tal interrogante, es necesario conocer la evolución y tendencia de investigación en el continente. De esta forma, se podrían diseñar hojas de ruta basadas en evidencia que consideren la disponibilidad y calidad de la evidencia del continente latinoamericano, pero que se encuentren adaptadas al contexto de desarrollo de salud de las características propias de la población ^10^.

La bibliometría es una herramienta esencial para cuantificar y describir el comportamiento de la investigación científica, ampliamente utilizada en ciencias biomédicas ^11^. Esta permite calcular el impacto de la producción científica y visualizar las redes de colaboración y la forma en que se realiza y publica la investigación, lo cual se debe correlacionar con la calidad y sistematización de la evidencia, para valorar brechas, pluralismo y relevancia en investigación médica ^12^^-^^14^.

Previamente, se han realizado estudios bibliométricos sobre falla cardiaca en Latinoamérica, pero estos se limitan a describir la producción científica de un solo país ^15^. Entonces, se desconoce el crecimiento científico y las características asociadas de esta temática en nuestra región. Para proporcionar información más rigurosa y relevante en el contexto regional, que sirva de base para el diseño de futuros estudios y líneas de trabajo, el objetivo de este estudio consistió en valorar, de forma visual, descriptiva y bibliométrica, la investigación latinoamericana en falla cardíaca en los últimos 20 años.

## Materiales y métodos

### Diseño del estudio

Estudio bibliométrico.

### Base de datos

Scopus, la base de datos más extensa de literatura científica revisada por pares, se utilizó como fuente para este análisis ^16^. Actualmente, bajo la categoría de literatura médica, esta base de datos indexa más de 15 mil revistas ^17^. Además, en comparación con otros motores de búsqueda, índices citacionales y bases de datos de calidad media-alta, como PubMed, PubMed Central y Web of Science, Scopus cuenta con un mayor número de indexaciones de revistas biomédicas latinoamericanas, lo que facilita la identificación de evidencia relacionada con la pregunta de investigación ^18^.

### Estrategia de búsqueda

Se diseñó y ejecutó una búsqueda no sistemática para identificar artículos relacionados con la investigación en falla cardíaca, ya sea básica, traslacional, clínica, experimental u otros en filiaciones latinoamericanas. Para ello, se tuvo en cuenta la filiación reportada en los metadatos y corroborada por la publicación oficial a texto completo. La estrategia de búsqueda se construyó basada en términos MeSH y sinónimos en inglés (considerando que se publican de manera estandarizada tanto en inglés como en español). Antes de la ejecución de la estrategia definitiva, se llevó a cabo una prueba piloto. Luego de esa prueba se utilizó la siguiente estrategia: TITLE-ABS-KEY(“Heart Failure”) OR TITLE-ABS-KEY(“Cardiac Failure”) OR TITLE-ABS-KEY(“Heart Decompensation”) OR TITLE-ABS-KEY(“Right-Sided Heart Failure”) OR TITLE-ABS-KEY(“Right Sided Heart Failure”) OR TITLE-ABS-KEY(“Myocardial Failure”) OR TITLE-ABS-KEY(“Congestive Heart Failure”) OR TITLE-ABS-KEY(“Left-Sided Heart Failure”) OR TITLE-ABS-KEY(“Left Sided Heart Failure”) OR TITLE-ABS-KEY(“Diastolic Heart Failures”) OR TITLE-ABS-KEY(“Diastolic Heart Failure”) OR TITLE-ABS-KEY(“Systolic Heart Failures”) OR TITLE-ABS-KEY(“Systolic Heart Failure”) AND AFFILCOUNTRY (antigua AND barbuda) OR AFFILCOUNTRY (argentina) OR AFFILCOUNTRY (bahamas) OR AFFILCOUNTRY (barbados) OR AFFILCOUNTRY (belice) OR AFFILCOUNTRY (bolivia) OR AFFILCOUNTRY (brazil) OR AFFILCOUNTRY (chile) OR AFFILCOUNTRY (colombia) OR AFFILCOUNTRY (costa AND rica) OR AFFILCOUNTRY (cuba) OR AFFILCOUNTRY (dominicana) OR AFFILCOUNTRY (ecuador) OR AFFILCOUNTRY (el AND salvador) OR AFFILCOUNTRY (grenada) OR AFFILCOUNTRY (guatemala) OR AFFILCOUNTRY (guyana) OR AFFILCOUNTRY (haiti) OR AFFILCOUNTRY (honduras) OR AFFILCOUNTRY (jamaica) OR AFFILCOUNTRY (mexico) OR AFFILCOUNTRY (nicaragua) OR AFFILCOUNTRY (panama) OR AFFILCOUNTRY (paraguay) OR AFFILCOUNTRY (peru) OR AFFILCOUNTRY (dominican AND republic) OR AFFILCOUNTRY (saint AND lucia) OR AFFILCOUNTRY (suriname) OR AFFILCOUNTRY (trinidad AND tobago) OR AFFILCOUNTRY (uruguay) OR AFFILCOUNTRY (venezuela) AND PUBYEAR > 2002 AND PUBYEAR < 2024 AND ( LIMIT-TO ( SRCTYPE , “j” )). Así, se limitó la búsqueda a países latinoamericanos, documentos publicados en revistas revisadas por pares y publicados entre 2003 y 2023.

### Estandarización y recolección de datos

Aunque la búsqueda se diseñó en inglés, considerando que en Latinoamérica predominan el español y el portugués, se incluyeron artículos publicados en estos idiomas. Una vez realizada la búsqueda, se exportaron datos como el año de publicación, el título del artículo, detalles de la revista, tipo de artículo, palabras clave, filiaciones, detalles sobre autores, citaciones, colaboración científica y detalles sobre el proceso editorial y de publicación. Esta búsqueda se llevó a cabo hasta el 7 de octubre de 2023. Se incluyeron finalmente documentos publicados bajo el proceso estándar de revisión por pares, específicamente en revistas científicas, donde se identificó la participación de al menos una filiación latina. Se excluyó la literatura que no sigue el proceso de revisión por pares regular para la publicación en revistas científicas, como libros, series de libros, resúmenes y memorias de eventos científicos.

Luego de esta primera fase se realizó una revisión manual por parte de dos autores (G.B.M. y Y.A.P.J.) para eliminar duplicados y aquellos artículos no relacionados al tópico de interés, basados en título, resumen y palabras clave. La estandarización y revisión manual se ejecutó en Microsoft Office Excel 2016. Finalmente, en tercer lugar, tres autores (G.A.G.P., E.R.P. y Y.A.P.J.) realizaron otra revisión manual, para poder estandarizar los datos de las variables de interés, y reducir las discrepancias entre la forma en la que se registran los metadatos originalmente. Así, se reagruparon categorías, por ejemplo, todos los artículos originales, independientemente del diseño observacional o experimental, fueron categorizados como «Artículos»; de la misma forma, todas aquellas revisiones, independientemente de su abordaje (ya sea narrativa, sistemática o metaanálisis), fueron categorizadas como «Revisiones». Series de casos y casos reporte fueron categorizados como «Reporte de caso», y editoriales, cartas al editor, comentarios, entre otros, fueron categorizados como «Correspondencias». De la misma manera, para garantizar la especificidad en las afiliaciones latinas, se revisaron y corroboraron estas.

### Análisis estadístico, visual y bibliométrico

Para determinar y valorar las tendencias, características e impacto científico de la investigación latina en falla cardíaca, se emplearon métricas de redes y bibliométricas. Todos los documentos que cumplieran con los criterios de inclusión se incluyeron en el análisis global. Para la realización de este análisis se utilizó el paquete Bibliometrix de R, que permite calcular indicadores bibliométricos cuantitativos y visualizar los resultados (versión 4.3.1) ^19^. Sinónimos, errores, plurales y variantes se agruparon cuidadosamente para homogeneizar el análisis. Se estandarizaron palabras clave, autores e instituciones.

Además, se realizó un análisis descriptivo y caracterización de la producción científica encontrada, evaluando el crecimiento científico anual, el promedio de citaciones por año, la frecuencia de publicación e indicadores de impacto. Se caracterizaron los autores más prolíficos y la distribución de publicaciones mediante la Ley de Lotka. También se especificaron los estudios que han acumulado el mayor impacto sobre la investigación en falla cardíaca en Latinoamérica. Se construyeron redes de colaboración para determinar el grado de colaboración entre países de la región y fuera del continente.

Para medir el impacto de autores, instituciones y países, se utilizaron métricas como el índice h, índice g, índice m y el valor absoluto de citaciones acumuladas. Las definiciones y especificaciones del uso de estas métricas en estudios bibliométricos se describieron previamente ^20^^,^^21^. El cálculo de frecuencias y porcentajes se realizó mediante Microsoft Office Excel 2016.

### Aspectos éticos

Este estudio no requirió aprobación por parte de un comité de ética, ya que no involucró investigación en humanos, modelos biológicos o el uso de historiales médicos.

## Resultados

**Figura 1:**
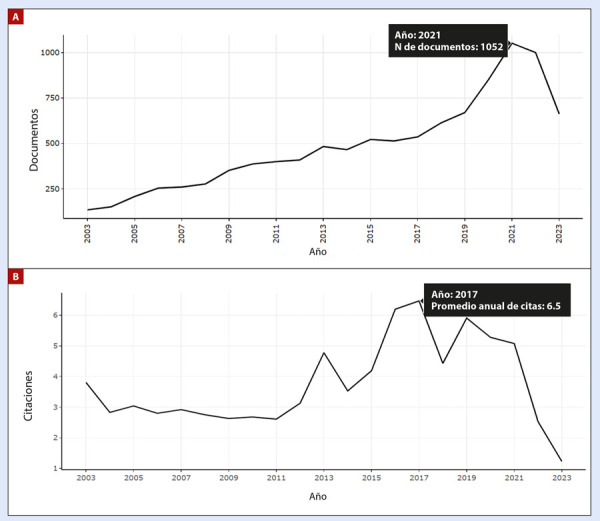
Crecimiento científico anual de la investigación en falla cardiaca en América Latina. A. Volumen de producción anual. B. Promedio anual de citas recibidas.

Inicialmente se identificaron 10 697 documentos. Posteriormente, tras la aplicación de criterios de inclusión y exclusión, se seleccionaron finalmente 10 204, abarcando el periodo de 2003 a 2023. De estos, el 66,9% (n=6824) fueron artículos originales, seguidos por artículos de revisión (15,5%; n=1,583). Se registraron un total de 66 940 autorías, de las cuales el 0,69% correspondió a autores con documentos de autoría única. La colaboración internacional representó el 38,41% (n=3919). La tasa promedio de crecimiento anual de la producción científica fue del 8,32%, y la media de citaciones recibidas fue de 30,75 **(**Tabla 1**)**. En la ventana de tiempo evaluada, se observó un crecimiento sostenido desde el año 2003 en la producción científica latina en falla cardíaca, alcanzando un pico de documentos (n=1052) en el año 2021, con un descenso hasta el 2023 **(**Figura 1A**)**. Una tendencia similar se observó en las citaciones recibidas, con un pico en el año 2017 (promedio de 6,5 citaciones) y una disminución dramática hasta el año 2023 **(**Figura 1B**)**.

**Tabla 1:** Características generales de la producción científica latina sobre falla cardiaca (N=10 204).

	n	%
Tipología de artículo		
Artículo original	6824	66,9
Revisión	1583	15,5
Caso reporte	1039	10,2
Correspondencias*	758	7,4
Autores		
Autorías	66 940	-
Autores de documentos con autoría única (N=66,940)	418	0,62
Colaboración		
Artículos con autoría única	527	5,16
Coautorías por artículo (media)	12,2	-
Coautoría internacional	3919	38,41
Palabras clave	13 117	-
Revistas	2025	-
Tasa de crecimiento anual	-	8,32
Edad promedio de artículo (años)	7	-
Promedio de citaciones por documento	30,75	-

*Incluye cartas al editor, editoriales, comentarios, etc.

**Figura 2:**
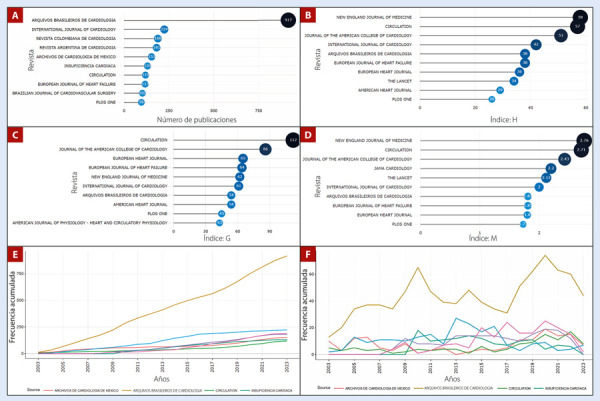
Evolución de las revistas e impacto de sus documentos sobre falla cardiaca, publicado por autores latinos. **A.** Frecuen cia de artículos publicados. **B.** Índice h de los artículos. **C.** Índice g de los artículos. **D.** Índice m de los artículos. **E.** Frecuencia acumulada de artículos publicados a lo largo del tiempo. **F.** Frecuencia anual de artículos publicados sobre falla cardiaca en Latinoamérica y el Caribe.

Al analizar las revistas de interés para la publicación de investigaciones sobre falla cardíaca por autores latinos, se encontró que Arquivos Brasileiros de Cardiologia (n=917; 8,9%), International Journal of Cardiology (n=224; 2,19%) y Revista Colombiana de Cardiología (n=118; 1,15%) poseen el mayor número de documentos repositados **(**Figura 2A**).** Por otro lado, al evaluar las revistas con el mayor impacto recibido por artículos publicados sobre falla cardíaca con autoría latina, se observa que New England Journal of Medicine (índice h = 58), Circulation (índice h = 57) y Journal of the American College of Cardiology (índice h = 51) lideran esta lista **(**Figura 2B**)**. Al medir los índices g y m, se destaca que Circulation (índice g = 112) **(**Figura 2C**)** y New England Journal of Medicine (índice m = 2,76) **(**Figura 2D**)** tienen las métricas más destacadas, respectivamente. New England Journal of Medicine es la revista con el mayor número de citas acumuladas (51 885 citas). Finalmente, al medir la ocurrencia acumulada de publicación entre las revistas de mayor interés para los autores, se observa un crecimiento sostenido entre el top 7, liderado por Arquivos Brasileiros de Cardiologia **(**Figura 2E**)**. A diferencia de la frecuencia de publicación anual, donde el comportamiento fue fluctuante, pero liderado por las mismas revistas de mayor interés **(**Figura 2F**)**. 

En relación con los 10 autores más prolíficos sobre falla cardíaca en la región, se revela que el 70% son brasileños, seguidos por un 20% con filiación argentina. Edimar Alcides Bocchi, de la Universidade de São Paulo (Brasil), y Rafael Díaz, del Instituto Cardiovascular de Rosario (Argentina), son los autores con el mayor número de artículos publicados sobre falla cardíaca, con frecuencias de 151 y 100, respectivamente. Sin embargo, el mayor impacto lo poseen Rafael Díaz y Alvaro Avezum (del Hospital Alemão Oswaldo Cruz, Brasil), quienes tienen índices h con valores de 48 y 45, respectivamente. Esta tendencia es similar en cuanto al número total de citaciones obtenidas y valores de índice m, donde estos dos últimos autores lideran estas métricas (21 155 y 18 099 citas, e índices m de 2,4 y 2,14, en el orden dado) **(**Tabla 2**)**. Además, al aplicar la Ley de Lotka, se evidencia que el 69,2% (n=46 305) del total de autores latinos identificados, solo ha publicado un artículo relacionado con falla cardíaca, mientras que el 17,1% (n=11 458) y 0,3% (n=168) han publicado al menos 2 y 10 artículos, respectivamente. 

**Tabla 2:** Resumen de autores, filiaciones y países latinos más prolíficos sobre investigación en falla cardiaca.

Autores	Documentos sobre falla cardiaca	Índice h	Índice g	Índice m	Total citaciones	Afiliación	País
Edimar Alcides Bocchi	151	31	54	1,47	3469	Universidade de São Paulo	Brasil
Rafael Díaz	100	48	100	2,4	21 155	Instituto Cardiovascular de Rosario	Argentina
Fernando Bacal	96	27	46	1,28	2401	Universidade de São Paulo	Brasil
Evandro Tinoco Mesquita	94	16	46	0,76	2206	Universidade Federal Fluminense	Brasil
Luis E Rohde	81	22	50	1,04	2588	Universidade Federal do Rio Grande do Sul	Brasil
Nadine Clausell	76	26	76	1,3	6413	Hospital de Clínicas de Porto Alegre	Brasil
Felipe Martínez	75	26	44	1,23	10 732	Universidad Nacional de Córdoba	Argentina
Charles Mady	74	19	34	0,9	1338	Universidade de São Paulo	Brasil
Alvaro Avezum	66	45	66	2,14	18 099	Hospital Alemão Oswaldo Cruz	Brasil
Sergio Lavandero	62	30	60	1,42	3677	Universidad de Chile	Chile
Afiliación	Documentos a lo largo del tiempo				Total documentos sobre falla cardiaca	Índice h	País
	2003 - 2007	2008 - 2012	2013 - 2017	2018 - 2023			
Universidade de São Paulo	606	1068	885	1071	3630	105	Brasil
Universidade Federal de São Paulo	89	166	240	259	754	55	Brasil
Universidade Federal de Minas Gerais	30	193	140	356	719	54	Brasil
Universidade Federal do Rio Grande do Sul	43	111	147	327	628	49	Brasil
Pontificia Universidad Católica de Chile	97	88	83	246	514	44	Chile
Universidade Federal Fluminense	35	156	118	169	478	24	Brasil
Instituto Nacional de Cardiología Ignacio Chávez	28	36	32	309	405	40	México
Universidad de Chile	67	93	68	109	337	38	Chile
Hospital Italiano de Buenos Aires	14	52	86	175	327	40	Argentina
Universidade Federal do Rio de Janeiro	30	58	106	89	283	44	Brasil
País	Documentos a lo largo del tiempo				Total documentos sobre falla cardiaca*	Índice h	SCP
	2003 - 2007	2008 - 2012	2013 - 2017	2018 - 2023			
Brasil	367	913	1211	2338	4829	182	3269
Argentina	88	231	348	720	1387	133	639
México	106	144	254	694	1198	91	612
Colombia	17	67	231	530	845	70	391
Chile	67	92	189	328	676	80	212
Perú	5	11	51	137	204	42	52
Cuba	21	31	39	83	174	16	92
Venezuela	34	40	46	42	162	35	36
Uruguay	14	18	42	62	136	31	37
Ecuador	1	6	16	82	105	24	18

*Se contó producción de forma individual. Por lo tanto, un documento pudo haberse contado varias veces en función de la colaboración internacional.

**Figura 3:**
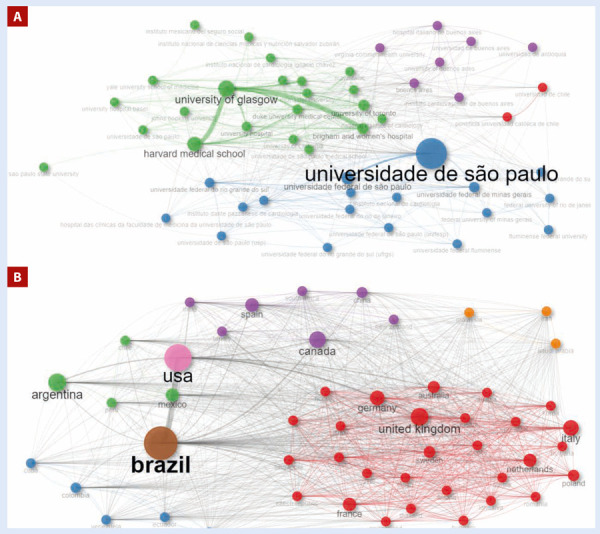
Redes de colaboración y fuerza de la colaboración regional e internacional de Lati noamérica en investigación en falla cardiaca. **A.** Colaboración de afiliaciones latinas. **B.** Fuerza de colaboración regional e internacional.

Al analizar las filiaciones más productivas sobre la temática, se determina que las tres instituciones más prolíficas son brasileñas, destacándose la Universidade de São Paulo significativamente en cuanto al número de documentos publicados e impacto obtenido (n=3630 e índice h = 105), seguido de la Universidade Federal de São Paulo (n=754 e índice h = 55) y la Universidade Federal de Minas Gerais (n=719 e índice h = 54) **(**Tabla 2**)**. Al valorar la transición en la producción desde el año 2003, las instituciones argentinas y mexicanas han experimentado un crecimiento lento en comparación con las brasileñas y chilenas, cuyo crecimiento ha sido más notable, especialmente en el periodo 2018-2023, donde muchas incluso duplicaron o triplicaron su producción **(**Tabla 2**)**. Al explorar la colaboración y la fuerza de colaboración de las instituciones, se observa que la Universidade de São Paulo, la más prolífica, tiene una red de colaboración fuerte y diversa, tanto a nivel regional como intercontinental, destacándose la colaboración con instituciones americanas como Harvard Medical School y Brigham and Women’s Hospital, así como con la University of Toronto en Canadá y la University of Glasgow en Escocia **(**Figura 3A**)**.

Al examinar el comportamiento de los países, se reconoce que Brasil (n=4829), Argentina (n=1387), México (n=1198), Colombia (n=845) y Chile (n=676) son los países más prolíficos de la región. En cuanto al impacto, Brasil (índice h = 182), Argentina (índice h = 133) y México (índice h = 91) lideran. A nivel global, la tendencia en el crecimiento de la producción ha sido similar, con un pico representativo a partir del año 2013, donde algunos países duplicaron su producción entre 2013-2017, y volvieron a duplicar este último valor entre 2018-2023 **(**Tabla 2**)**. En cuanto a la colaboración, es notable la extensa red que posee Brasil, con colaboraciones esenciales tanto con Estados Unidos de América y Canadá como con Argentina en Latinoamérica, y numerosos países europeos, incluso algunos asiáticos **(**Figura 3B**)**.

Al detallar los artículos sobre falla cardíaca con autoría latina (excluyendo estudios de colaboración masiva) que han obtenido el mayor impacto, se encuentran: 1) Effect of nesiritide in patients with acute decompensated heart failure (total citaciones: 1036; citaciones por año: 79,69; países participantes: Colombia, Brasil, Chile, Argentina, México; DOI: 10.1056/NEJMoa1100171); 2) Transendocardial, autologous bone marrow cell transplantation for severe, chronic ischemic heart failure (total citaciones: 1168; citaciones por año: 55,62; país participante: Brasil; DOI: 10.1161/01.CIR.0000070596.30552.8B); y 3) Heart failure: preventing disease and death worldwide (total citaciones: 813; citaciones por año: 8,3; país participante: Brasil; DOI: 10.1002/ehf2.12005). Todos estos fueron publicados en revistas de muy alto impacto, con colaboración de países de altos ingresos.

**Figura 4:**
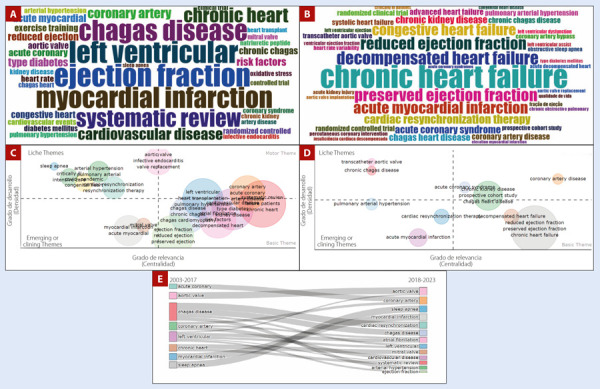
Tendencias en investigación en falla cardiaca. **A.** Nube de palabras clave en forma de bigramas más frecuentes. **B.** Nube de palabras clave en forma de trigramas más frecuentes. **C.** Mapa temático de los bigramas más relevantes. **D.** Mapa temático de los trigramas más relevantes. **E.** Transición de tendencias a lo largo del tiempo.

Finalmente, al examinar las tendencias en investigación sobre falla cardíaca en Latinoamérica, se observan hallazgos interesantes. Mediante nubes de palabras se encontró que la cardiomiopatía chagásica (n=413), la falla cardíaca asociada a infarto agudo de miocardio (n=296), revisiones sistemáticas (n=245), fracción de eyección y sus categorías (n=227), así como la falla cardíaca crónica (n=206), son algunos de los tópicos de estudio más frecuentes en la falla cardíaca latinoamericana **(**Figura 4A**y**4B**)**. Utilizando mapas temáticos se logró identificar que los temas fundamentales que constituyen preguntas de investigación están relacionados con la fracción de eyección, enfermedad crónica, factores de riesgo cardiovascular, descompensación y enfermedad de Chagas. Como tópicos emergentes o asociados a los temas fundamentales previamente descritos, se encuentran la apnea obstructiva del sueño, la terapia de resincronización, el reemplazo de válvula aórtica transcatéter e la hipertensión pulmonar **(**Figura 4C**y**4D**)**. Además, al dividir los abordajes de investigación que han sido de interés en la falla cardíaca en Latinoamérica entre los años 2003-2017 y 2018-2023, se evidencia que se mantienen un número significativo de tópicos, con la adición de interés en la sistematización de evidencia, el estudio de la fibrilación auricular y la resincronización cardíaca **(**Figura 4E**)**.

## Discusión

Este estudio representa el primer análisis en habla hispana que examina las tendencias y la evolución de la investigación y publicación científica en falla cardíaca en Latinoamérica durante un periodo de 20 años. Según datos de sociedades científicas e instituciones públicas latinoamericanas, la falla cardíaca es una de las enfermedades cardiovasculares más prevalentes en la región, existiendo necesidades importantes en la búsqueda de mejorar desenlaces e indicadores en salud asociados a esta condición ^22^^,^^23^. No obstante, no existe una hoja de ruta clara y específica para cada país, que adopte sus prioridades en función de la demanda y desenlaces en salud, que facilite el planteamiento y ejecución de proyectos de investigación, encaminados a responder eventuales preguntas problema pertinentes, y que impacten de forma real y positiva en la población. Por ejemplo, previo al diseño de una estrategia o política, es necesario conocer las brechas y relevancia en investigación médica, para determinar si es prioritaria la investigación clínica, social, traslacional o básica en falla cardiaca ^23^. Esto implica evaluar cuantitativamente la producción, los tópicos cubiertos y las áreas del conocimiento, así como identificar las instituciones e investigadores líderes, que son posibles colaboradores para impulsar la investigación colaborativa.

Del total de documentos analizados, se observó que predominantemente (66,9%), corresponden a estudios originales, subrayando el interés y avance en la producción de datos primarios en investigación en falla cardíaca. No obstante, en comparación con un estudio bibliométrico global reciente sobre falla cardíaca ^24^, que abarcó un período de 2009 a 2019 en Web of Science, se encontró que el total de documentos fue de 21 484. Aunque las ventanas de tiempo y las bases de datos evaluadas difieren, este hallazgo sugiere que la producción científica en Latinoamérica podría representar un porcentaje significativo de la producción global. Este fenómeno podría indicar que, a pesar de las dificultades en infraestructura, financiación y disponibilidad de talento humano exclusivamente dedicado a la investigación, la región ha progresado siguiendo las tendencias de investigación global en falla cardíaca. Este progreso se refleja en el alto porcentaje de colaboración internacional (aproximadamente 40%) y la baja proporción de autoría única (0,69%). Además, se observó una tasa de crecimiento anual cercana al 9%, con una media de 30 citaciones. A pesar de estos indicadores positivos, se notó una reducción notable en las citas recibidas en los últimos años, lo que puede deberse a un período corto entre la publicación y el análisis o a una disminución en el interés por estudios o artículos específicos en el discurso científico nacional e internacional. En comparación con otros análisis bibliométricos del mismo tema ^25^, que muestran un crecimiento sostenido hasta la actualidad, en Latinoamérica, el impacto demostrado por las citaciones ha disminuido, presentando un desafío para la evaluación continua de ideas y proyectos en falla cardíaca ^26^. Incluso, correlacionar si los abordajes y tipos de investigación se relacionan con las necesidades en ciencia y salud de la región.

En cuanto a las revistas científicas regionales de cardiología con mayor interés, se destacó que Arquivos Brasileiros de Cardiologia y Revista Colombiana de Cardiologia repositoron un número significativo de documentos. Esto sugiere que, en casos de investigación predominantemente local o regional, los autores pueden preferir revistas de su región o sus propias revistas ^27^. A pesar de ello, International Journal of Cardiology también tuvo un impacto relevante, indicando que la evidencia científica ha alcanzado el interés del discurso científico y académico global, dado que esta revista tiene un alto impacto. En contraste, basándonos en las métricas de impacto y citaciones, revistas multidisciplinarias en medicina como New England Journal of Medicine, Circulation y Journal of the American College of Cardiology lideran métricas. Esto puede explicarse por su interés en la publicación de ensayos controlados aleatorizados multicéntricos y estudios prospectivos a gran escala, cuyos datos y resultados tienen un potencial de validez externa significativo ^28^. En consecuencia, existen autores latinos que contribuyen a estos consorcios y aportan datos primarios de población latina, generando estudios de alto impacto. El crecimiento sostenido en este tema de investigación se refleja en el aumento de la producción de Arquivos Brasileiros de Cardiologia, que lideró los picos de publicación en el periodo evaluado. Este crecimiento también se correlaciona con la posición de liderazgo en investigación de la región que tiene Brasil, ya que esta revista es nacional.

A pesar de que Brasil, sus autores y universidades, especialmente la Universidade de São Paulo y la Universidade Federal de São Paulo, tienen una proporción significativa de la producción global identificada, también se destacan Argentina y México debido a algunos autores y filiaciones. Sin embargo, sería interesante profundizar en las subáreas o tópicos específicos en los que cada institución o país lidera, aunque este análisis está fuera del alcance de la metodología utilizada. A pesar de estas diferencias, significa que, a diferencia de Brasil, hay otros países que pueden ser contribuyentes y/o líderes potenciales en la investigación en algunas subáreas de falla cardíaca en Latinoamérica. Sin embargo, se necesita una dirección más clara por parte de instituciones y sociedades científicas líderes, ya que algunos países e instituciones han experimentado un crecimiento desproporcionado en los últimos años en comparación con otros en la región. Esto refleja la necesidad de impulsar e invertir más en ciencias de la salud ^29^, especialmente en condiciones de interés en salud pública como la falla cardíaca. La colaboración con países de altos ingresos, como Estados Unidos, Canadá y Escocia, podría ser la razón fundamental por la cual Brasil lidera la investigación en falla cardíaca en la región. Su red y fuerza de colaboración son mucho mayores en este campo de la ciencia en comparación con el resto de los países de Latinoamérica. Esta colaboración, ya sea a nivel nacional, regional o intercontinental, indudablemente puede impulsar macroproyectos de investigación, desarrollo e innovación ^29^. Ejemplos de esto son los estudios más citados y realizados en instituciones latinoamericanas (excluyendo ensayos y colaboraciones masivas), que fueron publicados en revistas de alto impacto y tienen métricas acordes con su relevancia científica a nivel internacional. 

Finalmente, al evaluar las tendencias y transición de las líneas de investigación en falla cardíaca desde el año 2003, se observó un cambio notable relacionado con la tendencia de medicina de precisión en los últimos años. Mientras que de 2003 a 2017 se estudiaron tópicos destinados a caracterizar, analizar e indagar sobre factores y patrones asociados a ciertas enfermedades cardiovasculares relacionadas con la falla cardíaca, en los últimos cinco años se ha intensificado la sistematización de datos y las pruebas de intervención para la resolución de la falla cardíaca. Este cambio es previsible, ya que después de un período significativo de producción de conocimiento sobre la población de interés, se busca sistematizar y calcular estimados que permitan determinar la precisión de una intervención y, finalmente, definir prácticas basadas en la evidencia aplicables a la población real ^30^^-^^32^.

Este estudio revela un panorama atractivo, original y relevante en la investigación en falla cardíaca, que puede ser utilizado por investigadores, grupos de investigación, instituciones estatales, universidades y sociedades científicas interesadas en evaluar lo que se ha investigado y lo que se debe investigar sobre la falla cardíaca. Considerando las brechas y el pluralismo en el conocimiento, así como las posibles colaboraciones basadas en instituciones e investigadores líderes en la región, el uso de recursos bibliométricos ^33^^-^^36^, es fundamental para diseñar una hoja de ruta basada en evidencia que considere la inversión, la producción y los resultados de indicadores en salud y ciencia.

En conclusión, este estudio identificó un crecimiento en la investigación en falla cardíaca en Latinoamérica en los últimos 20 años, con Brasil y algunas de sus universidades liderando esta tendencia. Argentina y México también se destacan por su impacto y pluralismo en investigación. La amplia colaboración, especialmente con países de altos ingresos, parece ser clave en el impulso y avance de la investigación en esta área, siendo Brasil el país más influyente en la región. Además, se observó un interés sostenido en algunos tópicos relacionados con las causas o patrones de falla cardíaca, con una transición reciente hacia un gran interés en la sistematización de datos y la terapia de resincronización.

## Fuente de financiamiento

Autofinanciado
